# Dr. Duggan

**Published:** 1913-12

**Authors:** 


Dr. Duggan.—We are gratified and pleased to announce that Dr. Duggan, our Eugenics editor, has entirely recovered from the trouble for which he sought treatment at Baltimore, and will assume his position on the Eugenics firing line at an early date. Meantime he is taking advantage of being at Johns Hopkins to attend the great clinics and take instruction under the great masters. Dr. Hull, Secretary of the State Association of Social Hygiene, is very ably filling Dr. Duggan's place as editor of the department, and has contributed an able and timely paper on "The Social Evil."

---

The occasional occurrence of subperiosteal fracture of the patella should be borne in mind as a possible explanation of continued disability after trauma or muscle violence. It is only one of the conditions that radiography may elucidate which other means of examination of the knee fail to reveal.—*American Journal of Surgery.*

